# Genomic characterization of an ESBL-producing *Klebsiella pneumoniae* ST147 recovered from a hospitalized patient in Armenia

**DOI:** 10.1128/spectrum.03773-25

**Published:** 2026-07-16

**Authors:** Siyu Lu, Jie Sheng, Mary M. Ter-Stepanyan, Yingxiong Wang, Hermine V. Mkrtchyan

**Affiliations:** 1 Department of Paediatrics, Second Affiliated Hospital of the Army Medical University 105785 , Chongqing, China; 2 School of Basic Medical Sciences, Chongqing Medical University 12550, Chongqing, China; 3 The Joint International Research Laboratory of Reproduction and Development, Ministry of Education 630616 , Chongqing, China; 4 Faculty of Public Health, Department of Epidemiology, Yerevan State Medical University after M. Heratsi 159228 , Yerevan, Republic of Armenia; 5 Research Center of Maternal and Child Health Protection 681231 , Yerevan, Armenia; 6 Centre for Innovation for Genomics and Microbiome Sciences, University of West London 7364, London, United Kingdom; Department of Medicine and Technological Innovation, Universita degli Studi dell'Insubria, Varese, Italy

**Keywords:** *Klebsiella pneumoniae*, whole-genome sequencing, ESBL-producing, ST147

## Abstract

*Klebsiella pneumoniae* sequence type 147 (ST147) is a globally disseminated multidrug-resistant (MDR) clone associated with hospital outbreaks, yet genomic data from low-income countries remain scarce. Here, we report the first genomic characterization of an extended-spectrum β-lactamase (ESBL)-producing *K. pneumoniae* ST147 isolate (ARM07) recovered from a hospitalized patient in Armenia. Antimicrobial susceptibility testing revealed resistance to multiple antibiotic classes, while genomic analysis identified seven antimicrobial resistance (AMR) genes, namely, *strA*, *strB*, *bla*^CTX-M-15^, *bla*^SHV-11^, *sul2*, *qnrS1*, and *catII.2,* consistent with its multidrug-resistant phenotype. Phylogenetic analysis showed that ARM07 clustered closely with nine Russian ST147 isolates, with divergence estimated around 2014 (95% CI: 2009 to 2018), suggesting a recent common ancestor. Compared with its Russian relatives, ARM07 carried fewer AMR and virulence determinants, implying lower selective pressure in Armenia. Nevertheless, ARM07 harbored a distinct plasmid profile, IncFIB(pKPHS1), IncR, IncFIA(pBK30683), and Col440I, and the insertion sequence (IS) *ISKpn19* adjacent to AMR genes, indicating ongoing genomic plasticity and potential for further resistance acquisition. Our findings highlight the importance of genomic surveillance in Armenia and similar settings for monitoring the emergence and spread of high-risk *K. pneumoniae* ST147 lineages, providing essential evidence to guide targeted antimicrobial stewardship and infection control strategies.

**IMPORTANCE:**

We report the first in-depth genomic analysis of an extended-spectrum β-lactamase (ESBL)-producing *Klebsiella pneumoniae* ST147 isolate (ARM07) recovered from a hospitalized patient in Armenia, a low- and middle-income country (LMIC) where genomic surveillance capacity remains severely limited. Antimicrobial susceptibility testing revealed resistance to multiple antibiotic classes, while genomic analysis identified seven antimicrobial resistance (AMR) genes, namely, strA, strB, blaCTX-M-15, blaSHV-11, sul2, qnrS1, and catII.2, consistent with its multidrug-resistant (MDR) phenotype. Phylogenetic analysis revealed that ARM07 shares a recent common ancestor with Russian ST147 isolates but has since diverged, possessing distinct AMR determinants, virulence features, and plasmid repertoire, likely shaped by local selective pressures. The identification of this high-risk clone in Armenia highlights the potential for regional dissemination of clinically important *K. pneumoniae* lineages in settings with limited genomic surveillance capacity.

## INTRODUCTION

*Klebsiella pneumoniae*, a gram-negative rod-shaped bacterium, has emerged as a major global health threat due to its increasing virulence and remarkable capacity for antibiotic resistance (1). Since the 1960s and 1970s, it has emerged as one of the leading pathogens responsible for hospital-acquired, drug-resistant infections worldwide, particularly causing urinary tract infections (2), respiratory tract infections (3), and bloodstream infections (4). As an opportunistic pathogen, *K. pneumoniae* is strongly associated with increased morbidity and mortality, especially among immunocompromised and hospitalized patients (5). Its ability to persist in healthcare environments and colonize medical devices has contributed to recurrent hospital outbreaks, thereby posing significant challenges to infection prevention and control strategies (6, 7). Through distinct evolutionary trajectories and the acquisition of diverse genetic elements, *K. pneumoniae* can be broadly classified into two major pathogenic lineages: multidrug-resistant *K. pneumoniae* (MDR-*Kp*) and hypervirulent *K. pneumoniae* (*hvKp*) (1, 8). Infections caused by MDR-*Kp* are typically described as “classical *K. pneumoniae*” infections and are notable for their rapid and widespread transmission, particularly in high-risk healthcare settings such as intensive care units (ICUs) (9, 10). Moreover, MDR-*Kp* has the capacity to acquire multiple antimicrobial resistance (AMR) determinants, substantially amplifying its clinical significance and public health impact.

*K. pneumoniae* is of particular concern as a prominent member of the ESKAPE group of multidrug-resistant pathogens and has been classified by the World Health Organization (WHO) as one of the top three priority pathogens for which new antibiotics are urgently needed (11). Alarmingly, MDR isolates have been increasingly reported worldwide, with *K. pneumoniae* accounting for nearly one-third of all gram-negative infections in healthcare settings (6, 12). The global prevalence of antimicrobial resistance in this pathogen has reached up to 70%, with mortality rates ranging from 40% to 70% (13). These alarming statistics highlight the urgent need to enhance surveillance efforts, develop effective therapeutic strategies, and deepen our understanding of the genetic mechanisms driving resistance and virulence in *K. pneumoniae*.

*Klebsiella pneumoniae* sequence type 147 (ST147) has emerged since the mid-2000s as one of the most widespread and clinically significant MDR-*Kp* lineages globally (14). First identified in Europe in 2008–2009 as a CTX-M-15-producing clone, ST147 has rapidly disseminated across continents, driven by fluoroquinolone resistance mutations (gyrA S83I and parC S80I) and the acquisition of diverse extended-spectrum β-lactamases (ESBLs) and carbapenemases, including *bla*^CTX-M-15^ (15), *bla*^KPC^ (16), *bla*^NDM^ (17), *bla*^VIM^ (18), *bla*^OXA-48^ (19), and *bla*^OXA-18^ (20). Its global expansion has been accelerated by plasmid-mediated horizontal gene transfer, high genomic plasticity, and extensive intercontinental movement. ST147 has been implicated in numerous nosocomial outbreaks and has been recovered from a wide range of clinical specimens (21), animals (22), and environmental sources (23). Comparative genomic analyses have demonstrated that ST147 forms several phylogenetic clades that are correlated with geographic distributions and resistance profiles, with notable evidence of frequent intercountry and intercontinental transmission events (24, 25). Although ST147 rarely harbors classical hypervirulence plasmids, the lineage is characterized by efficient colonization, high transmissibility, and substantial resistance burden, highlighting its significance as an emerging global public health concern (26, 27).

The most comprehensive genomic study to date examined 172 ST147 isolates collected from 38 countries across 5 continents between 2002 and 2019, providing critical insights into the evolutionary dynamics and global epidemiology of this high-risk clone (28). Despite its clinical relevance, data on its molecular epidemiology, virulence, and prevalence within this lineage remain scarce, highlighting the need for continued surveillance and genomic investigation. Whole-genome sequencing (WGS), an essential tool for monitoring pathogen outbreaks, has been widely adopted in high-income countries (29). However, in low- and middle-income countries (LMICs), including in Armenia, the implementation of WGS remains limited, resulting in a lack of data on AMR profiles and pathogen transmission pathways. To date, our team has reported WGS data on *K. pneumoniae* sequence types 967 (30), 307 (31), and 37 (32) from Armenia; however, no comparative genomic analyses of ST147 isolates have yet been conducted in Armenia, despite this lineage being a globally disseminated, high-risk clone associated with multidrug resistance. Addressing this knowledge gap is essential for understanding the genetic characteristics, evolutionary dynamics, and antimicrobial resistance determinants of *K. pneumoniae* circulating in Armenia, contextualizing these isolates within global phylogenomic frameworks, and incorporating these findings into a broader international context.

In this study, we report for the first time the WGS analysis of a *K. pneumoniae* ST147 isolate recovered from the sputum of a hospitalized patient in Armenia. Further comparative bioinformatics analyses revealed the genomic characteristics of this isolate and its evolutionary and phylogenetic relatedness to international ST147 lineages. Our findings highlight the importance of genomics-based surveillance for identifying regional variation within pathogen populations and demonstrate how genetic characterization of emerging high-risk clones can strengthen AMR surveillance and inform effective infection prevention strategies.

## RESULTS

### Antibiotic susceptibility testing of ARM07

Antimicrobial susceptibility testing revealed that ARM07 was resistant to eight antibiotics: β-lactam antibiotics ampicillin, piperacillin-tazobactam, and amoxicillin-clavulanic acid; the cephalosporin antibiotics cefepime and ceftazidime; the fluoroquinolones norfloxacin and levofloxacin; as well as chloramphenicol (Table S1). However, it remained susceptible to the aminoglycoside antibiotic amikacin and the carbapenem antibiotic meropenem. In addition, ARM07 showed intermediate resistance to the carbapenem antibiotic imipenem.

### Phylogenetic analysis of the global *K. pneumoniae* ST147 population

**Fig 1:**
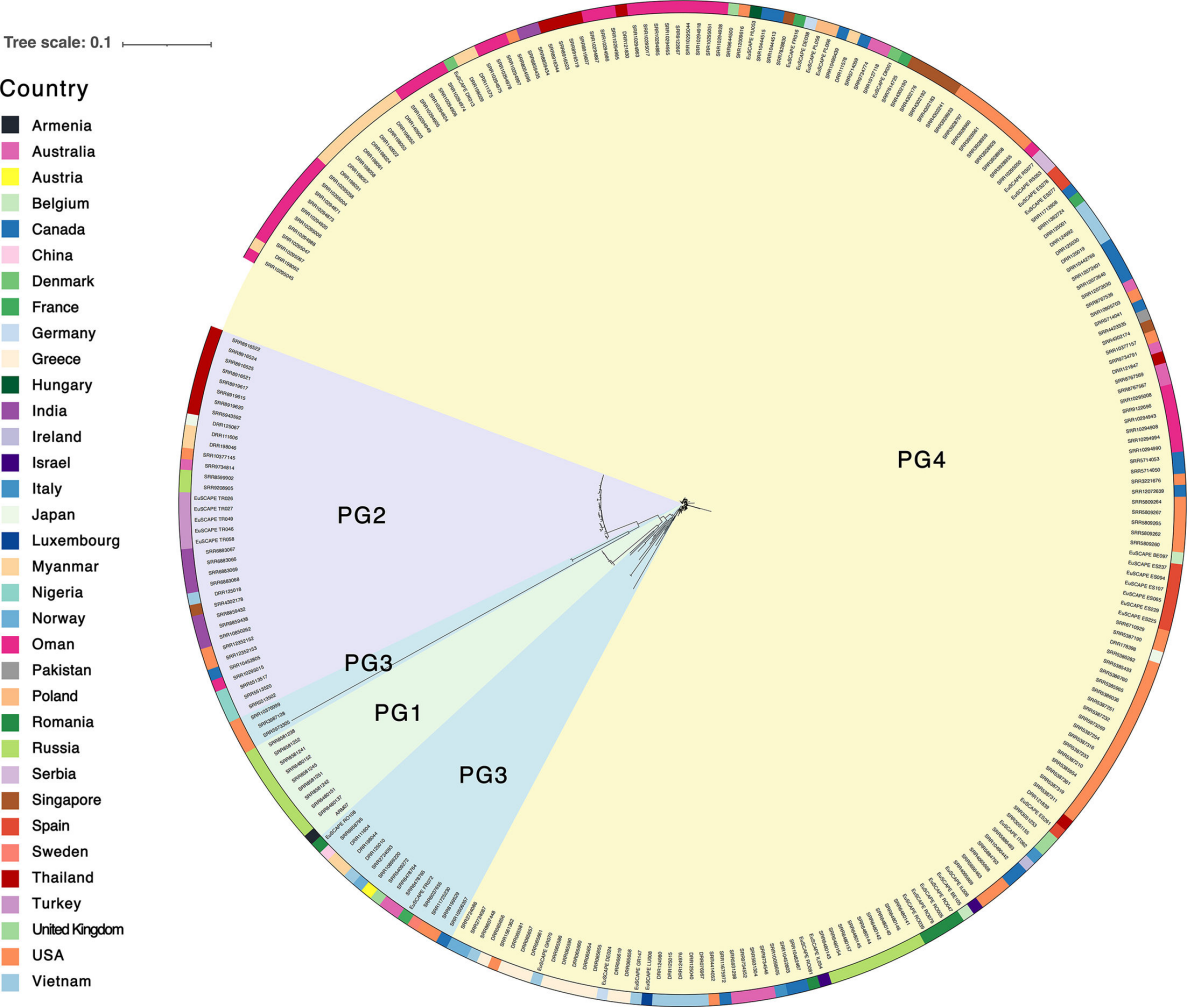
Global phylogenetic relationships of *K. pneumoniae* ST147 isolates based on core SNPs. Maximum-likelihood (ML) phylogeny inferred from core genome alignments, illustrating the global population structure. The tree is divided into four genetically distinct groups (PG1–PG4), as identified by RhierBAPS, with each group highlighted by a distinct color in the inner ring. The outer ring indicates the geographic origin of each isolate, with unique colors assigned to different countries.

Multilocus sequence typing (MLST) analysis revealed that *K. pneumoniae* ARM07 belongs to ST147 (Table 1). To further investigate the evolutionary relationship of ARM07, we performed a whole-genome core single-nucleotide polymorphism (SNP)-based phylogenetic analysis using 271 *K. pneumoniae* ST147 genomes obtained from the Pathogenwatch database (Fig. 1; Table S2). These genomes were originally recovered from 33 countries, the majority of which were collected from humans (Fig. S1). Only one isolate (SRR10899220) was recovered from a horse. The resulting phylogeny was divided into four phylogenetic groups (PGs), as defined by hierarchical Bayesian clustering using RhierBAPS. PG1 included 10 isolates, including ARM07. PG2, PG3, and PG4 contained 36, 18, and 208 isolates, respectively, all recovered from diverse geographic regions. Phylogenetic analysis revealed that PG4 was evolutionarily distant from the other three groups (PG1–PG3); therefore, our subsequent investigations focused primarily on PG1–PG3.

**TABLE 1:** Genomic characteristics of ARM07^*a*^

Antibiotics, virulence factors, or molecular data		Gene type or molecular data
Antibiotics		Antimicrobial resistance genes
Beta-lactams		
ESBLs		*bla* ^CTX-M-15^
Ampicillin		*bla* ^SHV-11^
Phenicols		*catII.2*
Fluoroquinolones		*qnrS1*
Sulfonamides		*sul2*
Aminoglycosides		*strA* and *strB*
Virulence-associated genetic elements		Virulence-associated genes
Hypermucoidy (rmpA and/or rmpA2)		–
Yersiniabactin (ybt)		–
Colibactin (clb)		–
Salmochelin (iro)		–
Aerobactin (iuc)		–
Others		*yagZ/ecpA*
Molecular data		
MLST		ST147
Capsule (K) locus		KL20
O antigen locus		O1/O2v1
Plasmid replicon		IncFIB(pKPHS1), IncR, IncFIA(pBK30683), and Col440I
Insertion sequence (IS)		*ISKpn19*, *ISKpn1*, *IS5075*, and *IS26*

^*a*^ ESBLs, extended-spectrum β lactamases; −, negative. Hypermucoidy (rmpA and/or rmpA2) represents the regulator of mucoid phenotype A.

### Genetic origins of *K. pneumoniae* ST147 isolates closely related to ARM07

**Fig 2:**
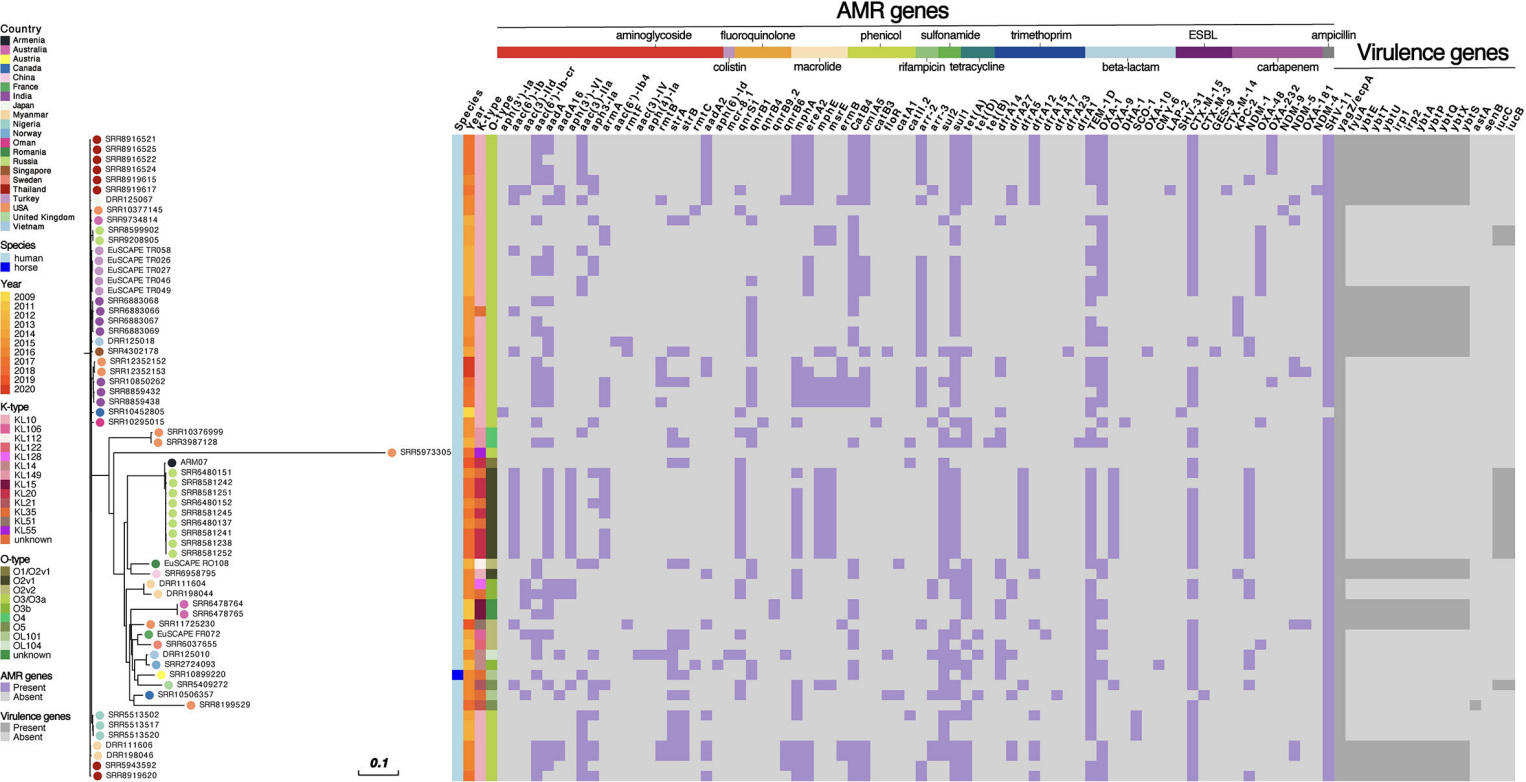
Detailed phylogeny and associated metadata of *K. pneumoniae* ST147 isolates within clades PG1–PG3**.** Core-genome maximum-likelihood phylogeny focusing on isolates closely related to the index strain ARM07. The country of isolation is indicated by the color of circles at branch tips. The adjacent heatmap displays key phenotypic and genotypic metadata for each isolate across six categories: (i) host species, (ii) year of collection, (iii) capsule (K) serotype, (iv) lipopolysaccharide (O) serotype, (v) presence (purple) or absence (light gray) of acquired AMR genes, and (vi) presence (dark gray) or absence (light gray) of selected virulence determinants.

A core SNP phylogenetic analysis was performed on 64 *K. pneumoniae* ST147 genomes representing phylogenetic groups PG1–PG3 (Fig. 2). Within PG2, the majority of isolates possessed the KL10 K antigen locus and the O3/O3a O antigen locus. The only exception was SRR6883066, for which the K antigen locus could not be determined. In contrast, no specific K or O antigen types were associated with PG1 or PG3. Notably, ARM07 harbored the KL20 capsular locus and O1/O2v1 antigen locus (Table 1). Phylogenetically, ARM07 clustered within PG1 and shared a close genetic relationship with nine isolates recovered from Russia.

**Fig 3:**
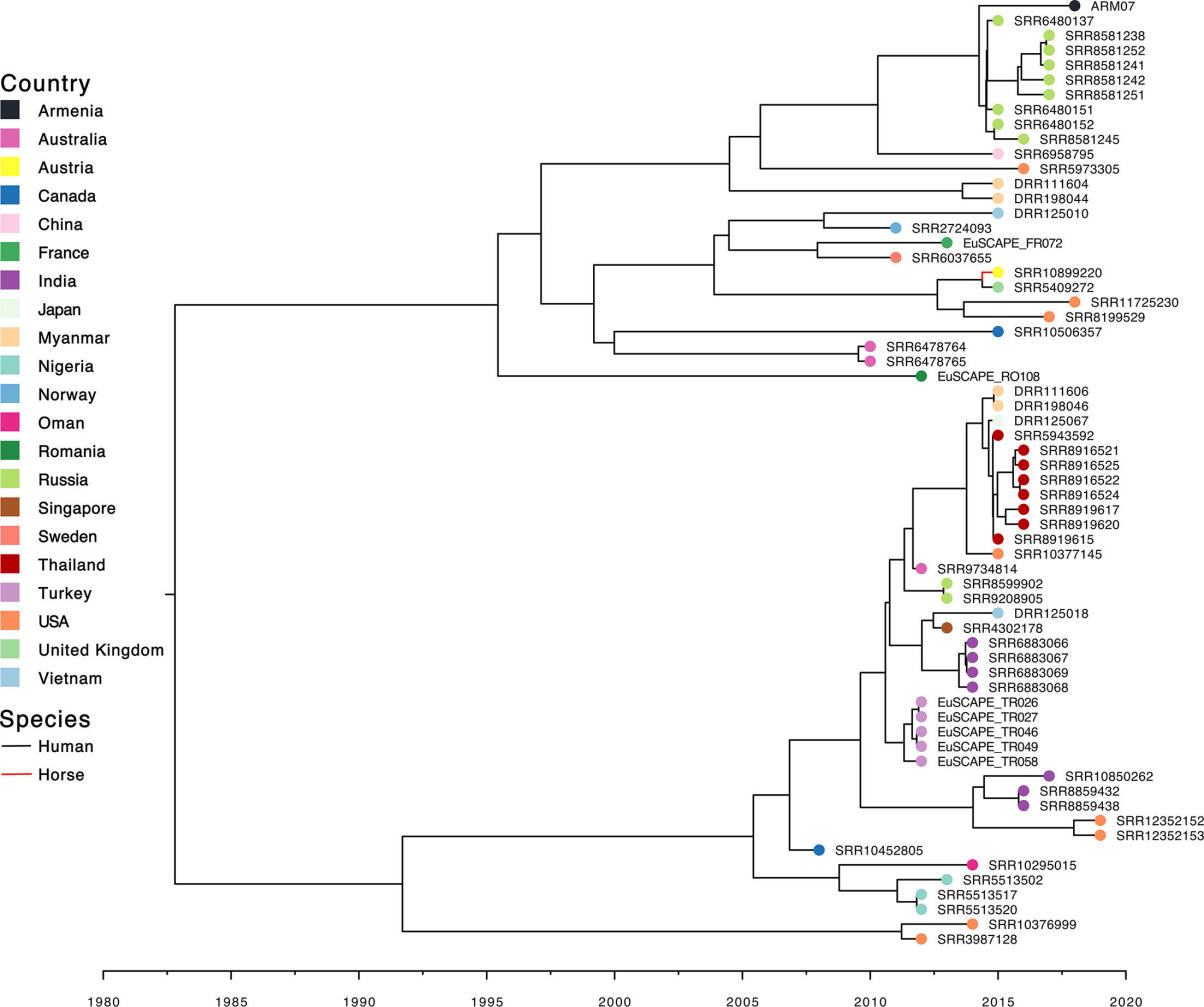
Temporal and phylogeographic analyses of *K. pneumoniae* ST147 isolates. Time-scaled MCC phylogeny reconstructed through Bayesian evolutionary analysis, with the timeline (Years) displayed at the bottom. Branch colors represent the host species of each isolate. Colored circles at the branch tips denote the country of origin for the corresponding strains.

We then reconstructed a maximum clade credibility (MCC) phylogenetic tree to investigate the evolutionary origin of ARM07 and its closely related ST147 isolates (Fig. 3). The MCC tree yielded an inferred root date of 1982 (95% CI: 1904 to 1995). The most recent inferred divergence between the Armenian isolate ARM07 and its nine closest phylogenetically related Russian isolates was dated to 2014 (95% CI: 2009 to 2018), suggesting that these isolates share a recent common ancestor. Average nucleotide identity (ANI) values derived from pairwise genomic comparisons further confirmed their high genetic relatedness (Fig. S2 ;Table S3). In addition, core-genome analysis revealed that ARM07 differs from the 9 Russian isolates by only 65 to 85 SNPs (Table S4). Collectively, the phylogenetic reconstruction, ANI analysis, and core-genome SNP comparison consistently support a recent divergence of ARM07 from the Russian ST147 isolates, likely occurring around 2014, from a shared common ancestor.

### Genotypes of resistance and virulence

A total of 74 distinct AMR genes were identified across the resistome of 64 isolates belonging to PG1–PG3, corresponding to an average of 13 AMR genes per isolate (ranging from 1 to 25) (Table S5). Several AMR genes were frequently detected, including the aminoglycoside resistance gene *aadA* (35/64), the macrolide resistance gene *mphA* (32/64), the phenicol resistance gene *catB4* (35/64), the sulfonamide resistance gene *sul1* (49/64), the β-lactam resistance genes *bla*^TEM-1D^ (49/64) and *bla*^OXA-1^ (36/64), the ESBL gene *bla*^CTX-M-15^ (50/64), and the ampicillin resistance gene *bla*^SHV-11^ (63/64). These findings indicate a high level of multidrug resistance among the isolates, with several clinically important AMR genes widely present across the population.

In contrast, ARM07 carried only seven AMR genes: the aminoglycoside resistance genes *strA* and *strB*, the fluoroquinolone resistance gene *qnrS1*, the phenicol resistance gene *catII.2*, the sulfonamide resistance gene *sul2*, the ESBL gene *bla*^CTX-M-15^, and the ampicillin resistance gene *bla*^SHV-11^ (Fig. 2; Table S5). Although the clade comprising nine Russian isolates was phylogenetically closest to ARM07, these isolates each harbored 14–17 AMR genes. Their AMR gene repertoire was highly conserved, with only minor differences observed in *aph(3')-Ia*, *armA*, *mphA*, and *bla*^CTX-M-15^. This discrepancy highlights the distinct AMR gene composition of ARM07, despite its close evolutionary relatedness to the Russian isolates, and suggests divergence in antimicrobial selection pressures or in the acquisition of resistance determinants via mobile genetic elements.

Next, we examined the virulence gene repertoires of all isolates in PG1–PG3 and found that all of them carried *yagZ/ecpA*, including ARM07 (Fig. 2; Table S6). Unlike ARM07, which harbored only *yagZ/ecpA*, its closest phylogenetic relatives (9 Russian isolates) additionally carried *iucB* and *iucC*. Among the other 23 isolates in PG1–PG3, all possessed *yagZ/ecpA* along with 11 yersiniabactin-associated genes (*irp1*, *irp2, ybtAEPQSTUX*, and *fyuA*). Furthermore, only one isolate, SRR8199529, recovered from the United States in 2018, was found to carry the *astA* gene. These findings indicate that ARM07 possesses a limited virulence gene repertoire compared to other PG1–PG3 isolates, suggesting potential differences in virulence capacity and ecological adaptation.

### Accessory genome and mobile genetic elements

**Fig 4:**
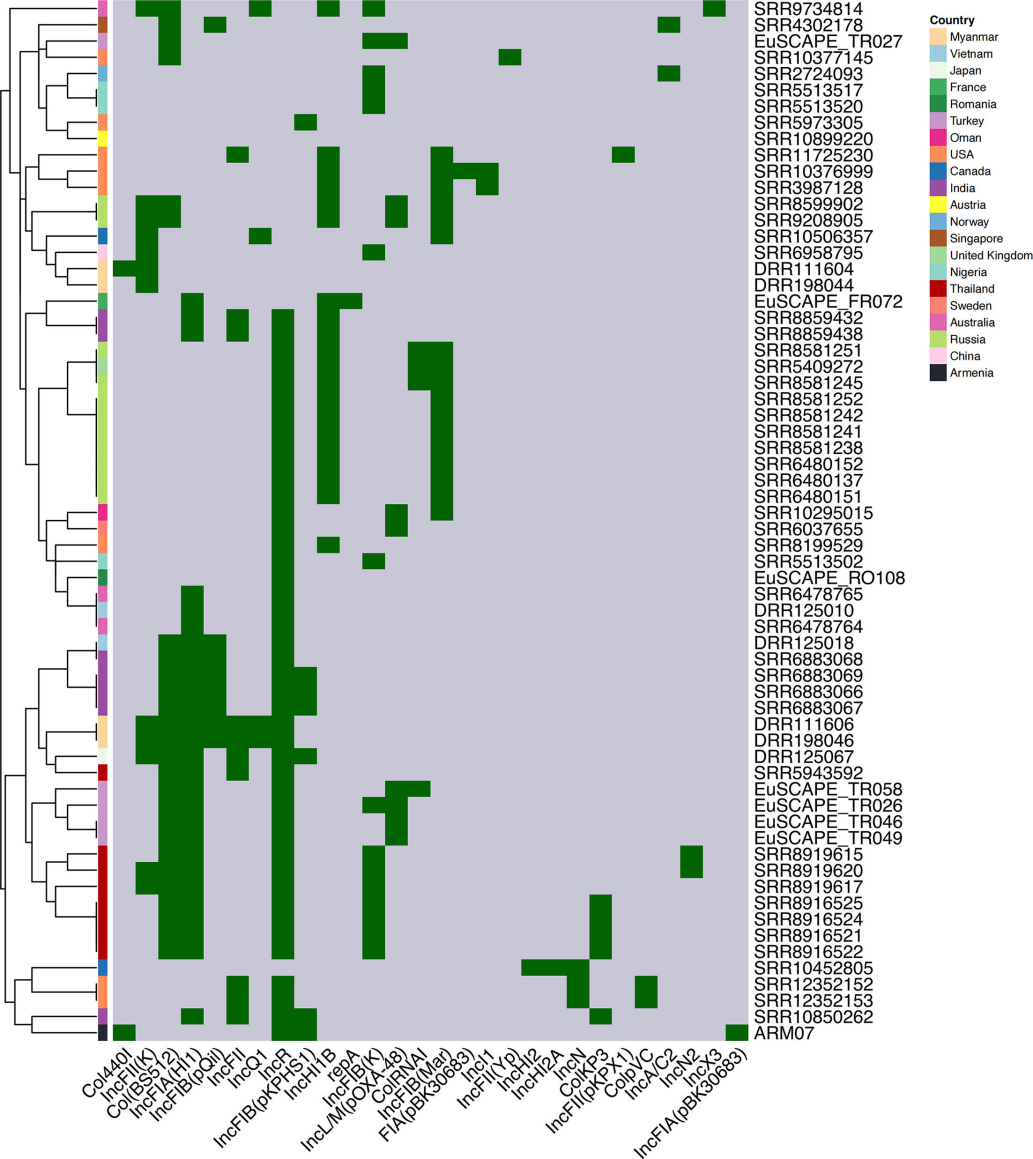
Plasmid replicon diversity and distribution among *K. pneumoniae* ST147 isolates. Presence/absence heatmap of plasmid replicon types, clustered hierarchically to group isolates with similar plasmid profiles. Each row represents an isolate, and each column represents a plasmid replicon type. Replicon presence is marked in green and absence in light gray. The colored annotation bar above the heatmap denotes the country of origin for each isolate.

Pan-genome analysis revealed a high correlation in the accessory genome between ARM07 and the nine Russian isolates, with Pearson correlation coefficients ranging from 0.897 to 0.913 (Fig. S3). A total of 28 plasmid replicons were identified among all *K. pneumoniae* ST147 isolates in PG1–PG3, with an average of three replicon types per isolate. Notably, ARM07 exhibited a unique plasmid replicon profile, harboring four replicons: IncFIB(pKPHS1), IncR, IncFIA(pBK30683), and Col440I (Fig. 4; Table S7). The *bla*^CTX-M-15^ gene (positions 2736–3611 on the contig) and the IncFIA(pBK30683) replicon (positions 5546–5840 on the contig) were colocalized on contig NODE_27. In contrast, all nine Russian isolates carried a conserved set of three plasmid replicons: IncFIB(Mar), IncHI1B, and IncR (Table S7). In addition, two Russian isolates (SRR8581245 and SRR8581251) also harbored ColRNAI, indicating minor plasmid variation within the group.

Furthermore, four ISs were identified in the ARM07 genome, namely, *ISKpn19*, *ISKpn1*, *IS5075*, and *IS26* (Fig. S4; Table S8). MOB-suite analysis predicted that *ISKpn1* was plasmid-associated. Notably, *ISKpn19* was located in close proximity to *strB* (approximately 115 bp apart) and resided on the same genomic fragment as *sul2*, *strA*, and *qnrS1*. Analysis of IS profiles showed that nine Russian ST147 isolates shared a conserved set of eight IS types, which included *ISKpn1* and *ISKpn19*, both of which were present in ARM07. Beyond SRR8581242, the Russian isolates additionally carried *IS6100* and ISEc9, elements that were absent in ARM07. *IS5075* was detected in all Russian strains except SRR6480137, while *IS26* was unique to ARM07 and two Russian isolates (SRR6480137 and SRR8581245). Together, these results indicate an expansion of mobile genetic elements in the Russian clade compared with ARM07. Using Scoary to assess the associations between accessory genes and strain-specific traits, we identified three genes uniquely present in ARM07: DNA adenine methylase (*dam*), D-allulose-6-phosphate 3-epimerase (*alsE*), and a putative protein YbiI (*ybiI*) (Table S9). These findings suggest that following divergence from a common ancestor, ARM07 and the Russian isolates likely underwent distinct horizontal gene transfer events driven by different selective pressures within their respective ecological or clinical environments.

## DISCUSSION

In this study, we report the first comprehensive genomic characterization of an ESBL-producing *K. pneumoniae* ST147 isolate (ARM07) recovered from a hospitalized patient in Armenia. Our findings address a critical knowledge gap in the current understanding of the molecular epidemiology of high-risk *K. pneumoniae* lineages in low-income countries, where systematic genomic surveillance is largely absent. By integrating phylogenetic analysis, resistome and virulome profiling, and the characterization of associated mobile genetic elements, we provide insights into the evolutionary dynamics and possible clinical implications of ST147 lineages circulating in Armenia.

Phylogenetic analysis revealed that ARM07 clustered closely with nine *K. pneumoniae* ST147 isolates from Russia, suggesting that these strains share a relatively recent common ancestor, with divergence estimated to have occurred around 2014. The short SNP distance of 65–85 between ARM07 and the Russian isolates, together with the high accessory genome similarity (Pearson correlation 89.7%–91.3%), indicates strong genomic relatedness across both the core and accessory genomes. This finding further supports the global transmission dynamics of *K. pneumoniae* ST147 and highlights the potential for intercountry transmission events. Recent studies have demonstrated nosocomial transmission of *K. pneumoniae* ST147 strains harboring unique *bla*^NDM-1^ introduced by Ukrainian medical evacuees in a Dutch pediatric oncology center, demonstrating how international patient movement can directly introduce multidrug-resistant pathogens into vulnerable, high-risk healthcare settings (33). Similarly, national surveillance carried out in Germany in 2022 reported a sharp increase in *bla*^NDM-1^- and *bla*^NDM-1/OXA-48^-producing *K. pneumoniae*, with genetic evidence linking many cases to patients from Ukraine and confirming subsequent local transmission (34). Together, these findings illustrate how clonal expansion coupled with transnational movement has facilitated the global spread of ST147. Importantly, the relatively recent divergence suggests that ST147 strains in Armenia and Russia originated from a shared ancestor introduced around 2014 and subsequently evolved under country-specific selective pressures. Differences in antibiotic usage, infection control, and healthcare infrastructure have likely shaped accessory genome variations, leading to modest divergence between the Armenian and Russian isolates. ARM07 showed resistance to several antibiotic classes, including β-lactams (ampicillin, piperacillin-tazobactam, and amoxicillin-clavulanic acid), fluoroquinolones (norfloxacin and levofloxacin), cephalosporins (cefepime and ceftazidime), and chloramphenicol, highlighting its multidrug resistance phenotype. Our results showed a strong correlation between AMR genes and resistance phenotypes: β-lactam resistance corresponded to the presence of *bla*^SHV-11^, fluoroquinolone resistance was associated with *qnrS1*, cephalosporin resistance was associated with *bla*^CTX-M-15^, and chloramphenicol resistance was associated with *catII.2*. The detection of the ESBL gene *bla*^CTX-M-15^ in ARM07 further confirms its resistance to extended-spectrum β-lactams, a trait commonly found in global ST147 strains (27). The largest genomic survey on ST147 reported that 86.6% of isolates carried *bla*^CTX-M^ (142/172), among which *bla*^CTX-M-15^ accounted for 93.7% (133/142) (28). Notably, *bla*^CTX-M-15^ is one of the most prevalent ESBL genes worldwide, often located on conjugative plasmids such as IncR (35), which greatly facilitates horizontal gene transfer and accelerates dissemination across different *Klebsiella* lineages and even other *Enterobacteriaceae* species (36). Its widespread distribution among ST147 highlights the strong selective advantage conferred by *bla*^CTX-M-15^ and highlights the importance of continued genomic surveillance to monitor its spread and guide targeted antimicrobial stewardship interventions.

Notably, ARM07 possessed a comparatively limited repertoire of AMR and virulence genes relative to its phylogenetically related Russian strains. Whereas the 9 Russian strains carried between 14 and 17 AMR genes, only 7 were identified in ARM07. Consistent with this reduced resistance gene repertoire, ARM07 remained susceptible to meropenem, distinguishing it from many globally disseminated ST147 isolates that frequently carry carbapenemase genes such as *bla*^KPC^, *bla*^NDM^, or *bla*^OXA^ (16–20). The marked difference in resistance gene carriage between ARM07 and the Russian isolates may reflect heterogeneity in local antimicrobial selection pressures, plasmid compositions, or the evolutionary trajectories of closely related ST147 lineages. In particular, the comparatively lower AMR burden observed in ARM07 may be associated with distinct antimicrobial usage patterns in Armenia, where reduced exposure to carbapenems could exert weaker selective pressure for the acquisition and maintenance of carbapenemase-encoding mobile genetic elements. Furthermore, the limited number of AMR genes in ARM07 suggests that this isolate may represent an earlier stage in the evolutionary progression of resistance within the ST147 lineage. Importantly, this does not preclude the future development of resistance. Indeed, the observed intermediate susceptibility to imipenem raises concern that carbapenem resistance may be emerging, highlighting the potential for ARM07 to acquire additional resistance determinants over time. Continued evolution of this lineage could, therefore, pose a significant clinical threat, highlighting the need for ongoing surveillance and robust antimicrobial stewardship.

Similarly, the virulence profile of ARM07 appeared comparatively limited compared to other ST147 isolates, such as those from Russia. ARM07 carried only the *yagZ*/*ecpA* gene, which is associated with biofilm formation. Biofilm formation is a critical factor in the persistence of infections, especially in healthcare settings, as biofilms enhance bacterial survival by providing protection against host immune defenses and antibiotics (37, 38). This makes infections more challenging to treat and increases their clinical relevance (37, 38). Notably, ARM07 lacked the yersiniabactin or aerobactin operons, indicating that ARM07 may have lower virulence potential than other ST147 lineages. This is consistent with previous reports that ST147 lineage generally lacks classical hypervirulence plasmids, instead relying on efficient colonization, environmental persistence, and transmission dynamics to maintain its epidemiological success (26, 27).

Despite a relatively limited virulence gene repertoire, ARM07 possessed a unique plasmid profile, including IncFIB(pKPHS1), IncR, IncFIA(pBK30683), and Col440I, all commonly associated with MDR loci that facilitate the dissemination of AMR genes (39, 40). The colocalization of *bla*^CTX-M-15^ and the IncFIA(pBK30683) replicon on contig NODE_27 provides direct genomic evidence that this clinically important ESBL gene is plasmid-borne. This physical linkage indicates that *bla*^CTX-M-15^ resides on an IncFIA-type plasmid, a replicon family frequently involved in the spread of antibiotic resistance among *Enterobacteriaceae*, thereby highlighting the potential role of IncFIA plasmids in shaping the epidemiology of CTX-M-15-mediated resistance. Moreover, IncFIA(pBK30683) has been reported to harbor *bla*^KPC-3^ and to possess conjugation functions enabling horizontal transfer among *Enterobacteriaceae* (41). IncFIB(pKPHS1) contains phage-like regions and can serve as a reservoir and vector for critical resistance genes, including the domestically prevalent *bla*^CTX-M-14^, via integration of mobile genetic elements such as Tn6558/Tn6559 (42). IncR plasmids, predominantly found in *Enterobacteriaceae* (e.g., *Escherichia coli*, *K. pneumoniae*, and *Enterobacter cloacae*), are highly stable and frequently carry MDR loci with diverse AMR genes and mobile genetic elements, promoting their accumulation and dissemination across gram-negative bacteria and posing a substantial global public health threat (43). Col440I, a class of small Col-type plasmids prevalent in *Enterobacteriaceae*, often carries resistance genes such as *qnrB*, facilitating the horizontal transfer of quinolone and other AMR determinants (44). Overall, this plasmid profile indicates that ARM07 carries multiple plasmids, which likely contribute to its multidrug resistance potential. The marked divergence in plasmid content between ARM07 and its nine closest Russian relatives highlights the high fluidity of plasmid-mediated gene exchange and supports the hypothesis that geographically distinct plasmid pools may drive local adaptive trajectories.

Moreover, the presence of the insertion sequence *ISKpn19* in proximity to AMR genes indicates ongoing genomic plasticity and a heightened potential for further acquisition or mobilization of additional resistance determinants. *ISKpn19*, an IS5 family insertion sequence associated with antimicrobial resistance, has previously been reported to colocalize with multiple resistance genes, including *qnrS1* (45), *bla*^CTX-M-15^ (46), and *bla*^NDM^ (47).

### Conclusion

Our findings emphasize that although ARM07 shares a recent evolutionary lineage with Russian isolates, it has undergone distinct divergence in antimicrobial resistance profiles, virulence determinants, and plasmid composition, likely driven by localized selective pressures. Importantly, the identification of an ESBL-producing ST147 isolate in Armenia highlights the potential for further dissemination of high-risk *K. pneumoniae* clones in regions with limited genomic surveillance infrastructure. Despite the limited sample size, our findings highlight the critical need to enhance pathogen genomics capacity in low- and middle-income countries to enable early detection, comprehensive monitoring of AMR trends, and the implementation of targeted, evidence-based infection prevention and control strategies.

## MATERIALS AND METHODS

### Antibiotic susceptibility testing

ARM07 was isolated from the sputum of a patient in Armenia in July 2019 and was one of eight *K. pneumoniae* isolates obtained from the Medical Microbiology Laboratories and reported previously (30). Species identification was performed using matrix-assisted laser desorption ionization–time-of-flight mass spectrometry (MALDI-TOF MS), as previously described (48). Subsequently, the antimicrobial susceptibility of ARM07 was assessed against 11 antibiotics commonly used in clinical practice in Armenia, following the guidelines of the European Committee on Antimicrobial Susceptibility Testing (EUCAST) protocol (49).

### Whole-genome sequencing

Genomic DNA was extracted as described in our previous study (30). Whole-genome sequencing was performed using the Illumina HiSeq platform. The quality of raw sequencing reads was assessed with FastQC v0.11.9 (https://github.com/s-andrews/FastQC). Reads were subsequently trimmed using Trimmomatic v0.38 (50), and quality-filtered reads were *de novo-*assembled using SPAdes v3.9 (51). Genome annotation was carried out with Prokka v1.14.5 (52).

### Multilocus sequence typing and molecular analysis

The sequence type of ARM07 was determined by analyzing sequence variations in seven housekeeping genes using the PubMLST database (https://pubmlst.org) (53). Molecular features, including AMR genes and K- and O-antigen loci, were identified using Kleborate v0.3.0 (https://github.com/katholt/Kleborate) (54). Virulence gene profiling was performed using Abricate v1.0.1 (https://github.com/tseemann/abricate) with the Virulence Factor Database (VFDB) (55). Plasmid replicon types were identified by querying the PlasmidFinder database using Staramr v0.7.2 (https://github.com/phac-nml/staramr) (56). Insertion sequences were detected using the MGE database (https://cge.food.dtu.dk/services/MobileElementFinder/). MOB-suite v3.0.1 (https://github.com/phac-nml/mob-suite) was used to predict plasmid and chromosomal contigs (57).

### Phylogenetics and pan-genome analysis

*K. pneumoniae* ST147 genomes from other countries were downloaded from the Pathogenwatch database (https://pathogen.watch) (58). To construct a core SNP-based ML phylogenetic tree of the *K. pneumoniae* ST147 isolates, all genomes were aligned against a reference *K. pneumoniae* ST147 genome (GenBank accession no. GCA_016903735.1). SNPs were identified using Snippy v4.6.0 (https://github.com/tseemann/snippy). Recombination regions were removed from the aligned sequences using Gubbins v2.4.1 (59). A phylogenetic tree was then constructed using FastTree v2.1.11 (60) with 100 bootstrap replicates under the GTR+GAMMA model and subsequently visualized and annotated using iTOL (61). Population structure analysis was performed using RhierBAPS with default parameters (62). Pan-genome analysis was carried out using Roary (63). Unique genes specific to the ARM07 isolate were identified using Scoary based on gene presence/absence data (64). Pairwise SNP distances between isolates were calculated using snp-dists (https://github.com/tseemann/snp-dists), and pairwise ANI was computed based on whole-genome sequences using the Python module Pyani v0.2.11 (65).

### Bayesian Evolutionary Analysis Sampling Trees (BEAST) analysis

A time-calibrated phylogenetic reconstruction of isolates in PG2–PG4 was performed using the BEAST software (66). The best-fit nucleotide substitution model was determined using jModelTest v2.1.10 (67). A Bayesian skyline coalescent prior, the GTR+Gamma substitution model, and a relaxed lognormal molecular clock model were applied. A Markov chain Monte Carlo (MCMC) analysis was performed over 10 million generations, with trees sampled every 1,000 generations. LogCombiner was used to merge the output files from five independent runs, applying a 10% burn-in. An MCC tree was then generated from the combined trees using TreeAnnotator (https://beast.community/treeannotator) and visualized with FigTree v1.4.4 (http://tree.bio.ed.ac.uk/software/figtree/).

## Data Availability

The short-read sequencing data have been deposited in the European Nucleotide Archive (ENA) under accession number ERR9882338. Accession numbers for all *K. pneumoniae* ST147 sequence files are listed in Table S2.

## SUPPLEMENTAL MATERIAL

Figure S1 spectrum.03773-25-s0001.tif Proportional distribution of *K. pneumoniae* ST147 isolates by country of origin.

Figure S2 spectrum.03773-25-s0002.tif Assessment of genome-wide relatedness based on ANI.

Figure S3 spectrum.03773-25-s0003.tif Hierarchical clustering heatmap of the accessory genome of *K. pneumoniae* ST147 isolates based on Pearson correlation.

Figure S4 spectrum.03773-25-s0004.tif Comparative analysis of insertion sequences in ARM07 and closely related ST147 isolates.

Supplemental tables spectrum.03773-25-s0005.xlsx Tables S1 to S9.

ASM does not own the copyrights to Supplemental Material that may be linked to, or accessed through, an article. The authors have granted ASM a non-exclusive, world-wide license to publish the Supplemental Material files. Please contact the corresponding author directly for reuse.
